# Exploring client violence during home visits: a qualitative study of perceptions and experiences of Israeli nurses

**DOI:** 10.1186/s13584-024-00640-w

**Published:** 2024-09-27

**Authors:** Yael Sela, Keren Grinberg, Inbal Halevi Hochwald

**Affiliations:** 1 Department of Nursing Sciences, Faculty of Social and Community Sciences, Ruppin Academic Center , Emeq Hefer, Israel; 2 Community Nurse, Maccabi Healthcare Services, HaSharon District, Israel; 3 School of Nursing, Max Stern Yezreel Valley College, Emek Yezreel, Israel

**Keywords:** Client violence, Home visit, Nurses, Organizational support, Physical violence, Risk, Verbal abuse

## Abstract

**Background:**

Home care provides an excellent opportunity for personalizing treatment as nurses see patients in their natural environment. Along with its many advantages, the home care environment carries unique risks, as nurses are usually alone, without the protection and security provided by primary care clinics. There are no accurate data in Israel on the scope and characteristics of client violence against nurses during home visits. We conducted a qualitative study to investigate the nature of client violence faced by Israeli nurses during home visits, to gain insights into their perceptions and experiences, and to contribute to the development of effective policies and strategies to combat client violence in the healthcare sector.

**Methods:**

Twenty-seven female nurses from primary care clinics, who were exposed to client violence during a home visit, were interviewed using a semi-structured interview guide. The interviews were transcribed and analyzed, and categories and themes were extracted.

**Results:**

Most nurses interviewed experienced at least three incidents of client violence, the most common of which was verbal abuse. The nurses perceived that the location of the encounter between the nurse and the patient in the patient’s natural surroundings, rather than within the controlled boundaries of a clinic, contributes to the risk of violence. Violence affected the nurses’ professional decisions. The nurses reported that their organization had no established guidelines or instructions for safely conducting home visits, they were not provided with protective or security measures for emergencies, nor did they perceive that they had sufficient training to deal with client violence in clients’ homes.

**Conclusions:**

Nurses encounter a range of challenges that make it difficult for them to deal with client violence during home visits, affecting their personal safety and professional decisions. Their ability to manage such situations is shaped by a complex interplay of personal and organizational factors and requires a range of strategies and resources to effectively address them.

## Introduction

Home care provides an excellent opportunity for personalizing treatment as nurses see patients in their natural environment [1]. It offers numerous benefits, including improved patient comfort, personalized care, cost-effectiveness, reduced hospitalizations, and enhanced patient satisfaction [2, 3]. Many patients and their families also prefer home-based care [1, 4]. The COVID-19 pandemic had expedited the transition from hospital care to home-based care, presenting a chance to reconsider the approach to providing healthcare in domestic settings [5].

Along with its many advantages, the home care environment carries unique risks that differ from those of nurses who work in clinics. In patients’ homes, nurses are visitors in an unfamiliar environment. They are usually alone without the protection and security provided by the primary care clinic. Many studies have reported that nurses experience violence in patients’ homes both by patients and their families [6–8]. Workplace violence against healthcare workers is defined as “incidents where staff are abused, threatened or assaulted in circumstances related to their work, including commuting to and from work, involving an explicit or implicit challenge to their safety, well-being or health” [7]. Workplace violence includes physical assault, aggression, sexual harassment, bullying, and verbal abuse or threats and can consist of a single incidence or multiple ones [9]. Workplace violence against care workers by patients or their families is sometimes referred to as “client violence” (also sometimes termed Type II violence [10], or third party violence [9]).

Healthcare workers have a 16-times higher risk for experiencing client violence than any other service profession [11]. More than a fifth of workers in homecare (22.3%) reported being subject to workplace violence and 30% of carers had experienced violence during their career [6]. Many visiting nurses worldwide have reported that they have been exposed to client violence in home settings [7, 12–14]. The most common form of client aggression is verbal abuse, experienced by 33-87% of home care personnel [15–18]. The prevalence of physical attacks (threats and actual attacks) on clinical healthcare personnel ranges from 17 to 74% [15, 18, 19].

Nurses who experienced violence in patients’ homes reported feeling stress, humiliation, mood changes, exhaustion, burnout, and lack of motivation [13]. Moreover, this affected their functioning and subsequently the quality of service provided to patients. In addition, absence from work and high employee turnover were reported [16, 20, 21]. The extent of the phenomenon and its serious consequences, has prompted professional organizations of nurses around the world, in collaboration with the World Health Organization, to issue guidelines and suggestions for strategies to deal with workplace violence [22]. They all share the understanding that as part of their responsibility to nurses, health organizations must initiate programs that will allow nurses to provide appropriate services in a safe and protected work environment.

Community nurses in Israel constitute about a third of all registered nurses in the country [23, 24]. Community and primary care nurses work mostly in one of the 4 health maintenance organizations (HMOs, also termed “health plans” or “health funds” in other publications on the Israeli health system), which provide health services under the National Health Insurance Law (1994) [25]. HMOs are required to provide medical and nursing services to patients in their homes according to their medical and functional condition. These services are provided by healthcare multi-professional teams which include physicians, nurses, psychologists, dieticians, and social workers [23]. As part of the services provided by HMOs, nurses make home visits after their working hours, providing wound care, vaccinations, intravenous fluids and medications [23, 26, 27].

There are no accurate data in Israel on the scope and characteristics of client violence against nurses during home visits and information about the risks associated with home visits is limited. The Director General of the Israel Ministry of Health has issued a circular outlining how to deal with violence against healthcare staff; however, it did not specifically refer to the unique risks in patients’ homes and how to deal with them [28]. Moreover, a published report on violence towards medical staff in the context of community care, only briefly referred to the patient’s home as a possible therapeutic arena [29].

This lack of information on client violence towards nurses during home visits has led us to initiate this study. We set out to (1) describe the scope of exposure to client violence among primary care nurses who make home visits, the types of violence they encountered, and their conduct during and after violent incidents; (2) to understand the perceptions and experiences of nurses who were subject to client violence during home visits and how these incidents affected their personal and professional lives; and (3) to examine how health organizations deal with incidents of client violence during home visits. Our ultimate aim was to contribute to body of knowledge on client violence during home visits in order to assist in the development of effective policies and strategies to combat client violence in the healthcare sector.

## Methods

### Study design

This qualitative study utilized a descriptive qualitative approach, involving in-depth semi-structured interviews analyzed through thematic content analysis [30]. This methodology was chosen because an interview about violence is personal and requires detailed descriptions, especially if the nurse had not reported it. The study design is appropriate for exploring questions related to the “who, what, and where” of events or experiences and for gaining a comprehensive understanding of nurses’ experiences with violence during home visits. The study was reviewed and approved by the institutional ethics committee. Prior to the interview, the interviewee’s consent was obtained, and assurances were given regarding anonymity.

### Participants

The study participants were 27 community nurses working in primary care clinics of three of the four HMOs in Israel. As the study aimed to collect personal and difficult accounts from the participants, which require a high level of trust and familiarity [31], potential participants were personally approached by the researchers via telephone or email. To be eligible to participate in the study, potential participants had to be registered nurses (RN), to work at a primary care clinic, to have conducted at least two home visits per week in the year preceding the study, and to have experienced at least one violent situation during a home visit. Primary care nurses who made less than 2 home visits in the preceding year and those who were not subject to client violence during home visits were excluded from the study.

### Interview guide

The semi-structured interview guide was developed following a literature review and consultation with three experienced senior managers working at the Israeli Ministry of Health. The first part included closed-ended sociodemographic questions. In the second part the interviewer presented the interviewees with an extensive definition of the term “violence”, including verbal abuse (shouting, swearing, threats) and physical violence (throwing objects or harming the nurse’s physical space or direct harm to the nurse, such as slapping, pushing, or hitting). The nurses were asked to evaluate the number of incidents they had experienced during the previous year and to describe who were the assaulters and how they perceived the reasons for the incidence. The third part focused on the impact of client violence during home visits on nurses’ professionalism and personal life, organizational support and process of handling home-visit violent incidents, and prevention and coping strategies with violence during home visits described in the second part.

### Data collection

The semi-structured in-depth interviews were conducted by a research team led by a female researcher with a PhD and experience in qualitative methodology. The researcher also works as a nurse in primary care clinics and had made home visits. The interviews took place from June to October 2021. Prior to the interview, the interviewee’s consent was obtained, and assurances were given regarding anonymity. The researcher knew most of the participants professionally prior to the study. Due to the sensitivity of the research subject, this was perceived as an advantage since the interviewees could open up and speak freely. The interviews were conducted according to the interview guide, lasted approximately 60 min and were held in the interviewee’s preferred location, either at a coffee shop or at the clinic. Additional questions emerged in an inductive, structured, evolving process [32]. The interviews were conducted until saturation was reached, as determined by the researchers [33, 34]. Only the researcher and the interviewee were present during the interview. Interviews were recorded and transcribed later [35]. The researcher took notes during the interview and immediately after the interview. Transcripts were not returned to the participants and no repeat interviews were conducted.

### Data analysis

An inductive content analysis process was used [36]. The Interview transcripts were read meticulously to identify common features. The findings were classified into content worlds that were repeatedly mentioned by the interviewees. These were classified into categories and then grouped into main themes and sub-themes, which served as the basis for the theoretical processing of the data [37]. To ensure the trustworthiness of the themes, two researchers reviewed the transcripts independently [38]. Issues, meanings, and strategies were considered themes when they appeared organically in most transcripts, and these were confirmed through detailed reflexive discussion among the researchers. Each category and interpretive claim were repeatedly checked and developed through re-scanning of the transcripts in search of examples, exceptions, variety, and nuance [34]. Each interpretive statement was accompanied by an illustrative verbatim quote [39]. The findings were summarized alongside the existing theory and literature, demonstrating whether the information has complemented or supplemented current knowledge.

## Results

### Characteristics of the study population

The study included 27 female primary care nurses, with a mean age of 42 years, of which 22 (80%) were married. All nurses had a bachelor’s degree in nursing and were registered nurses, 24 (89%) held a master’s degree in nursing, and three (11%) had a PhD degree. On average, the nurses had 15 years of experience working as community nurses in primary care clinics, with a range of 2 to 25 years of professional experience (Table 1). The participants worked in three of the four Israeli HMOs.

**Table 1:** Characteristics of the study population

Variables	Study population *N* = 27
**Age**,** years**,** mean (range)**	42 (24–57)
**Female**,** n (%)**	27 (100%)
**Education**,** n (%)**	
Bachelor’s degree/Registered Nurse	27 (100%)
Master’s degree	24 (89%)
PhD degree	3 (11%)
**Work experience**,** years**,** mean (range)**	15 (2–25)

### Characteristics of client violence during home visits

Twenty-one of the 27 nurses who participated in the study (78%) reported experiencing more than three violent incidents, while 6 (22%) reported one to three incidents. Verbal abuse was the most common form of violence experienced by most nurses (24/27 89%), whereas physical violence was reported by only three nurses (11%). Eighteen nurses (66%) reported that family members present during the home visit were the assaulters, and the rest (9/27, 33%) reported that the patients themselves were violent towards them (Table 2).

**Table 2:** Characteristics of client violence incidents

Variables	Study population *N* = 27 *n* (%)
**Number of client violence incidents experienced by the nurse during home visits**	
1–2	21 (78%)
> 3	6 (22%)
**Type of violence**	
Verbal abuse	24 (89%)
Physical violence	3 (11%)
Physical and verbal abuse	3 (11%)
**Assaulters**	
The patient	9 (33%)
A family member	18 (66%)

### Qualitative results

**Fig. 1:**
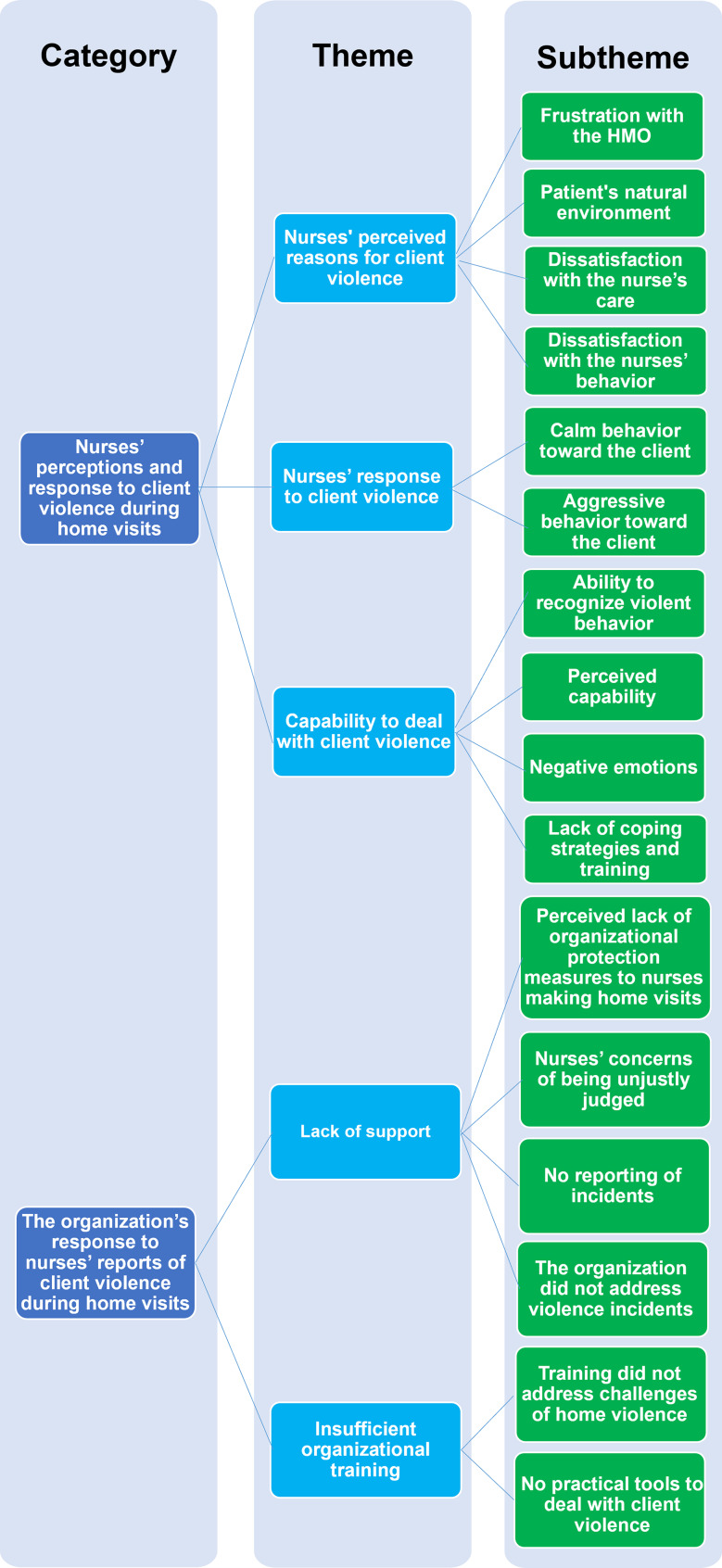
Categories, themes and subthemes revealed in the content analysis of nurses’ interviews on client violence during home visits

The interviewees provided an elaborate view of perspectives, challenges, and coping strategies with violence during home visits. The analysis revealed 2 categories, 5 themes and 16 subthemes (Fig. 1).

### Category 1: nurses’ perceptions and response to client violence during home visits

#### Nurses’ perceived reasons for client violence

Most nurses (24/27, 89%) perceived that the main reason for the violent behavior was frustration with the healthcare system and its services. They provided examples of patient complaints during home visits:

*Why didn’t they [the HMO] approve my medication?*

*Why does it take so long to make an appointment?*

*It’s because I don’t have contacts [at the HMO].*

They also mentioned that the ability to attack the nurse in their own home, within their natural environment was an enabler.

*The patient *i*s the host and [as such] has greater confidence*. *I’m sure that at the clinic, where he is a visitor, he reacts more cautiously.*

Only a few participants perceived that the reason for violence was dissatisfaction related to their professionalism or behavior at the time of the home visit.

*The bandage is too tight*.

*You don’t know what you’re doing*.

*I was a few minutes late. When I arrived the patient became very upset*,* saying that I was irresponsible and that his time was also valuable*,* and that his children had taken time off work specifically for this appointment and that I should reimburse them for the time they lost.*

In most incidents the presence of family members worsened the situation. While some family members attempted to calm the patient down, there were no incidents in which the patient attempted to calm down family members.

*His children were present during my visit…I remember that it was hot*,* and the windows were closed*,* and the patient complained about the wound dressing; and they stressed me out*,* to the point where my hands were shaking*.

*With his family around him the patient felt comfortable and confident enough to offend me*,* believing they would support him .*

### Nurses’ response to client violence

The nurses’ responses to aggressive behavior during home visits ranged from calm to aggressive attitudes. Some nurses reported trying to calm the aggressor by actively listening and using a quiet voice. However, when they felt that the conversation was not calming the patient sufficiently, and that the general atmosphere was intense, they hurried to complete the treatment and leave the house, or left without completing the treatment.

*First of all, as soon as he raised his voice, I listened to his complaints and tried to speak quietly to calm him down*.

*I tried to calm the patient, but I realized that nothing seemed to work, and I sensed that he was about to throw something at me. This had happened to me in the past, and at that moment I had a flashback of fear and I was overwhelmed by emotions. I simply ran out of the building, leaving all my belongings behind.*

Some nurses chose to please the patient, even at the cost of compromising their professionalism and quality of care.

*There was no need to withdraw the patient’s blood for testing*,* but because he insisted adamantly*,* I drew it and left quickly because I felt threatened.*

The nurses’ reaction was influenced by the home environment and the fact that they were alone and feeling vulnerable and exposed.

*I was aware that I was alone and that the situation could escalate quickly with no one to turn to, so I did my best to calm down although I realized I should have acted differently. In the clinic, I would have reacted assertively and confidently because I feel more secure, which might have made the situation more manageable.*

Only 3 nurses (11%) reported taking an assertive approach towards the attacker as soon as the incident began:

*I immediately responded in a loud and assertive tone*,* clearly stating that if they persisted in shouting or pressuring me*,* I would leave the house immediately.*

### Capability to deal with client violence

Overall, most participants (19/27, 70%) assessed their ability to deal with violence against them at the patient’s home as medium or below. They described feelings of helplessness and fear when facing a patient’s and/or family members’ aggression.

*When a person raises his voice and the tension escalates*,* I feel powerless*,* and completely out of control of the situation.*

It seemed that more experienced nurses estimated their ability to deal with client violence during home visits as higher than that of inexperienced nurses. Their past exposure to violence at the clinic helped them regulate negative emotions and react more calmly to stressful situations:

*Because of my experience with many different cases*,* I find that I can remain calm more easily and that I feel less stressed.*

*I believe I understand the patients’ perspectives and I don’t take things personally. I have always been a strong person. Maybe that has helped me not to take things personally and maintain assertiveness.*

Some interviewees had difficulty in recognizing and addressing violent incidents due to their perception of violent behavior. Some believed that such behavior was not intended to harm the nurse but was a result of the patient’s suffering and distress. Consequently, they adopted a forgiving attitude towards such events. Moreover, some interviewees, especially those with longer seniority, considered this behavior to be part of the professional risks that are inherent to nursing practice and even viewed it as a norm:

*I can empathize with the family; they did not receive a medication that could have saved their parent’s life. They are normative people who are currently experiencing distress and acting emotionally due to stress and anxiety.*

*Violence has always been present in our profession. It is just a part of the job. Nurses who have worked for many years have come to accept it as part of the job*,* especially in situations where a person is suffering.*

In response to violence or aggression, all participants reported an immediate and prolonged negative reaction characterized by shock, anger, and fear. Some nurses experienced sleep and eating disturbances, as well as concerns about their safety and that of their families. These emotions persisted for an extended period and impacted their professional functioning, as some nurses avoided being alone with patients at the clinic or while conducting home visits.

*I had an experience where a patient*,* who was a police officer*,* screamed at me and threatened to file a complaint that could cost me my job. His eyes looked menacing*,* which I still remember to this day. Afterwards I felt terrible for days. I felt traumatized and began to worry that since we live in the same community*,* he might track down me and my kids*.

*Since that incident*,* I find it difficult to function in the clinic. I feel compelled to overly accommodate patients*,* avoiding potential confrontations.*

One contributing factor to the participants’ sense of anxiety and lack of control during home visits was the absence of prior information about patients and their families. The interviewees noted that there was no available information about any prior incidents of violence or complaints of aggression, making it difficult for them to assess potential risks. Furthermore, it was challenging to infer the emotional state of the patient and their family solely based on the nature of the visit.

*Before the visit*,* I review the medical file*,* but there is no information about previous incidents of violence or [details about] his behavior and how they [such situations] were addressed.*

*Even if there is a psychiatric diagnosis in the patient’s file*,* what am I supposed to do [next]? Should I proceed with the visit? Ask for help? [Should I] review the patient’s entire mental history? There is no guidance on how to handle each case.*

### Category 2: the organization’s response to nurses’ reports of client violence during home visits

#### Lack of support from the organization

As for post-event management, 18/27 (67%) of interviewees said that the organization did not address or solve the problem of client violence. In addition, 15/27 (55%) said they did not have a helpful supervisor when they were exposed to violence.

Some nurses sought support from their organization to help them address the issue of violence during home visits. Most participants felt that the organization did not protect them or took special measures to protect them during home visits.

*If something happens to me*,* nobody will help me. No one knows where I am*,* and I don’t even know whom to call. The police? (laughing) That’s why sometimes I ask my husband to accompany me so that I won’t be alone if there is a problem.*

Several participants expressed distrust and frustration towards their direct managers and the healthcare system. While acknowledging the patient’s right to receive the care they deserve, the nurses were also concerned of the possibility of being unjustly judged and not receiving support for acts of violence or aggression held against them.

*No matter how much the patient may be at fault*,* at the end of the day*,* they are entitled to receive service without facing consequences for their actions. It is understandable that they file a complaint and make a big issue out of it… However*,* ultimately*,* I’ll end up being seen as guilty*,* which could significantly complicate my life.*

The interviewees’ perceptions that their reports would not receive support from the organization was evident from their failure to report incidents of violence and aggression. They believed that neither the authorities nor the organization would take any action against the aggressors. Consequently, none of the interviewees reported the incidents to the police or to the Ministry of Health. Moreover, only a small percentage of the interviewees (3/27, 11%) reported it to their direct manager, and that too only in cases of physical violence. The vast majority (24/27, 89%) did not report such incidents to the organization at all.

*Even when nurses experienced violence at the clinic, no actions were taken despite available measures, therefore, they would probably not address a case that occurred without witnesses*, *where it is essentially my word against the patient’s*.

### Insufficient organizational training about coping with client violence during home visits

During the interviews, the nurses were asked about their previous experience with training related to violence during home visits. Most of the nurses (24/27, 89%) reported that they had received some form of organizational training on violence, such as team meetings, seminars, or online courses, within the two years prior to the interview. Out of these nurses, two-thirds perceived that the training had a significant contribution in dealing with violence at community clinics, while one-third perceived it had a moderate contribution. However, all participants stated that the training did not address the specific challenges of violence during home visits, and that they were not provided with any practical tools or appropriate training to deal with such situations. They emphasized the need for a tailored training program adapted to the unique characteristics of home visits.

### Interviewer’s reflection

Working on this research brought back memories of incidents she herself had experienced during home visits. At the time, she did not realize these were instances of client violence, but in hindsight, it was clear they had been. This realization surprised her because, despite being an experienced and knowledgeable community nurse, she had not recognized her exposure to violence, nor had she addressed it or reported it to her managers and the organization. These reflections increased her sense of urgency to raise awareness and preparedness for such situations among nurses.

## Discussion

We have interviewed 27 nurses who experienced client violence during home visits.  Most had experienced at least three incidents of client violence, primarily verbal abuse and threats and, to a lesser rate - physical violence. Our findings are consistent with those of another study, conducted among 5506 Israeli healthcare workers, 26.4% of whom had experienced client violence during home visits, mostly verbal abuse; 2% reported physical violence [29]. It should be noted that client violence, and particularly sexual violence, is often under-reported [40, 41], raising the concern that the scope of the phenomenon is much wider.

The nurses estimated that the main reason for patients’ and family members’ violent behavior during home visits was anger and dissatisfaction directed at the bureaucracy of the health system and not necessarily directly related to the treatment or the nurse’s conduct. In another study nurses perceived that the meeting allows patients to ventilate their feelings of anger towards the system [42]. In the Israeli study mentioned above [29], unapproved medications and lack of reimbursement by the system were described as motives for violence against community healthcare staff but the nurses in our study estimated that the fact that the encounter with the nurse takes place in the natural surroundings of the patient, rather than within the controlled boundaries of a clinic or organization, is a contributing factor to the risk of being exposed to violence. The likelihood of emotional outbursts is greater in the home setting, as interactions between the patient, their family, and the nurse take place in the patient’s natural environment [16, 17, 21]. Lack of proper communication skills, withholding information from the family of a patient and lack of experience may also contribute to client violence [43, 44].

Furthermore, this “foreign” environment that is the patient’s home affected the nurses’ professional decisions and their response, so that in many cases, unlike decisions and actions they would take within the confines of the clinic, they chose to “please” the patient even if it did not comply with their professional perceptions, or they left the house before completing the visit. This gap can be explained by the fact that nurses have additional response options at the clinic, as shown by a study that examined how staff members react during violent incident at community and consulting clinics in Israel. Although in more than half of the cases (57%) the victim’s response was to try and calm the offender, the teams also chose other actions depending on the nature of the incident and their feelings, including calling the police (14%) or calling for help/activating an alarm (12%). In about 10% of cases, staff members reported that they called the clinic’s director and in a third of the cases, they reported that someone from the staff proactively came to help the victim [45]. In a similar Israeli survey, which was carried out a decade later, similar reports were received [29].

The nurses reported that their organization had no established guidelines or instructions for safely conducting home visits, they were not provided with protective or security measures for emergencies, nor did they feel that they had sufficient training that would help them restrain the patient and prevent the incident from escalating through a conversation. Insufficient organizational protection, together with a lack of protective measures and a low ability to cope appropriately were also reported by nurses who make home visits in other countries [42, 46–48]. Training that would have provided tools for developing a dialogue to restrain the patient’s behavior, alongside protective measures could have improved the nurses’ perceived competence to deal with the incident [49]. Security measures, such as a distress button, might have had a positive effect on the nurses’ professional decisions. In addition, all nurses interviewed estimated that dedicated training is required to prepare nurses to specifically deal with client violence in a foreign environment without the support of additional staff members. Without such training, nurses’ actions depend on their experience and on their personal traits.

The nurses did not report the incidents to a designated person in the organization. They mainly felt distrust that the organization would not take any action. Some nurses reasoned that the patient has a mental health issue. Home care aides experienced a higher risk of verbal abuse when patients had dementia, mental illness, psychological disorders, or limited mobility [50]. Other nurses accepted violence, especially verbal abuse, as an inherent risk to their work. Dunsford [51] has suggested that nurses perceive the risk of harm from a potentially violent situation as reasonable according to their experience and sense of duty to provide care. Thus, the perception of a situation as violent differs among nurses. Without appropriate training nurses may not recognize a violent incident.

Studies have also found that nurses rarely reported episodes of violence or verbal abuse to the police or to their organization since they believed nothing would be changed, supervised or solved [52]. Some nurses felt that they would not be believed if they complained about the incident – that it would be “their word against the client’s”. This underscores the intricate dynamics and tensions that often arise in healthcare settings when dealing with violence and aggression. According to the Israeli Law for Prevention of Violence in Treating Institutions − 2011, if a nurse reports a violent incident to his/her supervisor, the institution’s medical director can take sanctions against the aggressor, including ordering that he/she be prevented from entering the said institution for a period of up to six months, but the director cannot withhold medical treatment. As this sanction cannot be enforced in patients’ homes, it contributes to the nurses’ perceptions that no significant actions would be taken against patients who were violent during home visits. In Israel only 2% of violent incidents against healthcare personnel ended with legal charges against the aggressor. That is, out of approximately 3,000 incidents per year in Israel, 800–1000 cases are reported, of which only 8–10 cases reach a conclusion, usually long after the date of the incident [53].

The exposure and involvement in violent incidents and the perceived lack of organizational support negatively affected the nurses’ professional functioning as well as their personal life. Kim et al. reported that nurses were reluctant to provide services or became passive after being subject to violence [42].

Despite the importance of this issue, information on the prevalence of client violence against other healthcare professionals who make home visits, such as physicians, physiotherapists, and social workers is very limited. In a survey conducted in Australia among 529 general practitioners, 63.7% reported being subjected to some type of violence in the previous 12 months [19]. In another survey conducted in Australia among 168 general practitioners and other physicians who make after-hours house calls, 47.1% of the respondents had experienced at least one form of aggression over 12 months. The clients were aggressive towards the physicians in over half (51.8%) of the incidents, and family members and friends were the aggressors in 30.2% and 18.0% of the incidents. Most respondents expressed concern and apprehension about the risks of aggression (90.2% and 75.2%, respectively) [54]. In another survey, conducted in Germany among physicians, 33% and 39% of respondents reported experiencing client violence during house visits and house visits while on call, respectively, at least once during their career (14% and 16%, respectively, within the preceding 12 months), with verbal insults and threats being the most common type of violence. Among the respondents, 66% of females and 34% of males said they did not feel safe making house visits while on on-call duty [55]. Similar to our findings, and those of others, verbal abuse and threats were the most common form of aggression towards physicians making house visits [19, 54, 55].

### Strength and limitations

The study’s strength lies in its extensive assessment of the phenomenon from the viewpoints of nurses. The researcher knew most of the participants professionally prior to the study as she also works as a primary care nurse who makes home visits. Due to the sensitivity of the research subject, this was perceived as an advantage since the interviewees did not hesitate to answer the questions during the interview. As the interviewees work in 3 of the 4 Israeli HMOs, the study allowed to gain information on all but one HMO in Israel. However, the study population was limited to Hebrew-speaking nurses as the interviewer speaks only Hebrew. Nevertheless, as this is Israel’s official language, the majority of licensed nurses speak and understand Hebrew fluently. Only 27 nurses selected by specific criteria were interviewed, therefore, the results are limited to their views and therefore may not be generalizable to the entire population of Israeli nurses. Furthermore, it is worth noting that while qualitative studies are not generalizable in the same way as large quantitative studies, they allow for a more personal and detailed examination of phenomena from various perspectives. This depth of insight can contribute valuable knowledge to the field of study. Further studies on a larger sample may help quantify the phenomenon of client violence in home visits in Israel. To overcome a possible social desirability bias, during the interviews, in addition to assuring anonymity, the researcher listened actively without giving feedback, and emphasized that there are no correct or incorrect answers.

### Policy implications and recommendations

Governments and health services around the world regard reducing hospital admissions of older adults a major priority to improve healthcare outcomes and manage associated costs more effectively [56–59]. Therefore, alongside the growth in the aged population, the provision of healthcare services at home is expected to become more prevalent [60] together with incidents of client violence. In light of this trend, addressing client violence towards nurses and other healthcare professionals during home visits is crucial.

To address client violence effectively, changes must take place both nationally and within the organization, including in the units responsible for delivering home treatment. Nationally, a policy should be outlined by guidelines obliging HMOs to establish operative plans for protecting nurses during home visits. Second, similar to the possibility of prohibiting patients from visiting a clinic where they were violent, a policy should be formulated to apply sanctions to patients who are violent during home visits. For example, to impose the cost of sending several staff members or an accompanying security person on the violent client. Third. HMOs should be obliged to report incidents of violence during home visits in order to learn from these incidents and continue to update their policies accordingly.

The absence of organizational support is another major issue revealed by our study. Organizations must recognize this risk and establish procedures and support systems to deal with such situations in order to maintain the safety and wellbeing of their staff. Such support could include additional training and resources, access to counseling, and a commitment to implement stronger safety measures to protect staff members. An attendance reporting system must be developed that would allow the visiting nurse to notify her managers in advance of the time and location as well as the expected persons who will be present during the visit. This should be determined in a preliminary phone call with the patient and his/her family. Emergency call buttons should be installed on nurses’ mobile phones, which would enable calling emergency services for immediate help as well as direct communication with the nurse’s manager. Several organizations across different countries have adopted lone worker apps or monitoring systems as a means of improving the safety of their nursing staff. These systems enable nurses to alert their organization if they encounter a potentially hazardous situation while working alone [61]. According to Fazzone et al. [62], home visiting nurses perceived the possibility for direct and immediate communication with their employer during home visits as a lifeline in the event of a violent incident. In the Israeli health system, there are no procedures in place for home visits. Currently there is a mechanism for flagging violent patients in specific clinics’ visit systems; however, this is not applicable to home visits. Furthermore, this information is not regularly updated in the patient’s medical file, nor is it accessible to future healthcare providers conducting home visits with the same patient. Many home care visits are done by different healthcare providers over several days or months. Sharing information about incidents that had occurred at a patient’s home could be a key tool to increase awareness. Therefore, an internal reporting system that would only be visible to clinic staff should be established, to enable nurses preparing to make a home visit to get updates on prior abnormal or violent behavior of patients and their families both at the clinic and during home visits. The nurses in our study suggested that obtaining information in advance about patients’ medical condition could have facilitated their interactions during home visits. Furthermore, awareness of the phenomenon of client violence during home visits should be raised through the organization. Information on risk factors should be provided, together with operative tools and skills for managing violent situations with patients and their families. Managers should be trained to respond to nurses who have been affected by client violence during home visits. Actions reflecting a mindset of organizational legitimacy for learning from events are expected to lead to increased reporting of violent incidents.

At the level of the responsible unit, a departmental mechanism should be established for integrating all aspects of home visits and transferring relevant information among the unit nurses. Nurses making home visits should be monitored from the start to the end of the visit, so that their managers know where they are at every stage. In complex cases, a nurse with greater seniority and previous experience should make the home visit. As localities usually have several clinics that belong to the same HMO, establishing a team of home visit nurses specifically trained for the task should be considered.

Finally, a treatment contract between the client and the visiting nurse by which both parties commit to a set of behaviors related to the care of the patient is suggested [63]; however, at present there is insufficient evidence to suggest that this type of intervention is effective in preventing client violence in home settings.

Implementation of these recommendations should help reduce and event prevent violent incidents and their consequences. Moreover, although this study only focused on client violence against nurses during home visits, we believe that our findings and recommendations also apply to other health professionals who make home visits.

## Conclusions

Nurses encounter a range of challenges that make it difficult for them to deal with client violence during home visits. These include issues such as perceived justifications for client violence, lack of training in dealing with violent patients, inadequate safety measures, and limited access to patient history and background information. All these factors contribute to an overall sense of vulnerability and unease when conducting home visits. The ability of nurses to manage violence during home visits is shaped by a complex interplay of personal and organizational factors and requires a range of strategies and resources to effectively address them.

## Data Availability

The data supporting the results are available from the corresponding authors upon reasonable request.

## Abbreviations

HMO: Health Maintenance Organization
